# DarkASDNet: Classification of ASD on Functional MRI Using Deep Neural Network

**DOI:** 10.3389/fninf.2021.635657

**Published:** 2021-06-24

**Authors:** Md Shale Ahammed, Sijie Niu, Md Rishad Ahmed, Jiwen Dong, Xizhan Gao, Yuehui Chen

**Affiliations:** ^1^Shandong Provincial Key Laboratory of Network Based Intelligent Computing, University of Jinan, Jinan, China; ^2^École de Technologie Supérieure (ÉTS), Montreal, QC, Canada

**Keywords:** autism spectrum disorder, fMRI, neuroimaging, image processing, deep learning, DarkASDNet, ABIDE

## Abstract

Non-invasive whole-brain scans aid the diagnosis of neuropsychiatric disorder diseases such as autism, dementia, and brain cancer. The assessable analysis for autism spectrum disorders (ASD) is rationally challenging due to the limitations of publicly available datasets. For diagnostic or prognostic tools, functional Magnetic Resonance Imaging (fMRI) exposed affirmation to the biomarkers in neuroimaging research because of fMRI pickup inherent connectivity between the brain and regions. There are profound studies in ASD with introducing machine learning or deep learning methods that have manifested advanced steps for ASD predictions based on fMRI data. However, utmost antecedent models have an inadequacy in their capacity to manipulate performance metrics such as accuracy, precision, recall, and F1-score. To overcome these problems, we proposed an avant-garde DarkASDNet, which has the competence to extract features from a lower level to a higher level and bring out promising results. In this work, we considered 3D fMRI data to predict binary classification between ASD and typical control (TC). Firstly, we pre-processed the 3D fMRI data by adopting proper slice time correction and normalization. Then, we introduced a novel DarkASDNet which surpassed the benchmark accuracy for the classification of ASD. Our model's outcomes unveil that our proposed method established state-of-the-art accuracy of 94.70% to classify ASD vs. TC in ABIDE-I, NYU dataset. Finally, we contemplated our model by performing evaluation metrics including precision, recall, F1-score, ROC curve, and AUC score, and legitimize by distinguishing with recent literature descriptions to vindicate our outcomes. The proposed DarkASDNet architecture provides a novel benchmark approach for ASD classification using fMRI processed data.

## 1. Introduction

**Figure 1:**
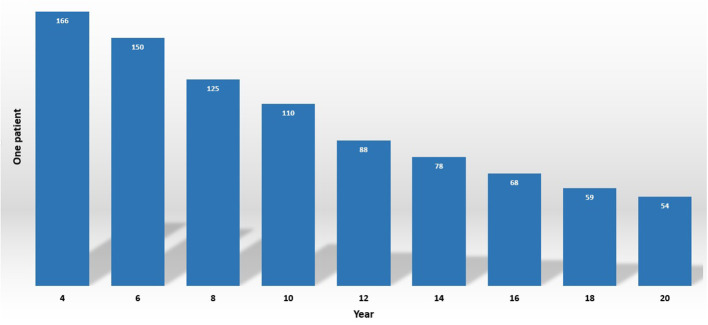
Estimated ASD prevalence of 2020 by CDC.

Autism spectrum disorder (ASD) is also familiar as a “spectrum” disorder that can cause different abnormalities such as social deficits, repetitive behaviors, speech, and nonverbal communication (Baio et al., 2018; Noriega, 2019). The fact-finding for the frequency of ASD is estimated at about 1% or higher (1 subject in 54, Figure 1) by the Center for Disease Control and Prevention in the United States (Senn, 2020). Previous treatments are based on the behavior observations of the patients, and the doctor asks a lot of psychological questions to the patient or their parents or guardians (Höfer et al., 2017; Hyman et al., 2020). These questionnaires often produce a false positive rate. The principal goal of neuroscience research is to sort out brain disorder treatment in an effective way (Yahata et al., 2016; Ahmed et al., 2018). Nevertheless, when patients seek a doctor for their treatment, sometimes the diagnosis of ASD is burdensome due to a lack of proper symptoms and the process requiring too much time (Mandell et al., 2007; Nylander et al., 2013). In consequence, it is indispensable to come up with conscientious techniques that can easily get make the diagnosis ASD more meticulous and efficient in an assessable way beyond depending utterly on behavioral questions.

The increasing investigation of neuroimaging research using up-to-date technologies in the last few years led to the classification of ASD, resulting in more effective performance in treatment (Bi et al., 2018). With the help of fMRIs, we can inspect the abnormalities between ASD vs. TC by analyzing functional connectivity (Kaiser et al., 2010; Lee et al., 2018). After introducing machine learning (ML) in neuroimaging, it becomes a legitimate means to obtain information from the raw data to illustrate the pattern of the disease (Klöppel et al., 2012). Amongst the ML approaches, in the area of neuroimaging research, support vector machines (SVM) is a powerful classifier to classify the problems (Sundermann et al., 2014; Chen et al., 2016).

Region of interest (ROI) bestows the structural medium, quantifying connectivities within the individual brain's active functional patterns. Many researchers investigate ASD individuals based on data-driven strategies or brain parcelation, such as independent component analysis (ICA), clustering, and dictionary learning by adopting ROI techniques (Cociu et al., 2018; Bi et al., 2019). Although the ROI strategy has some limitations regarding the arbitrary decision and standardization, considering the special regions can be biased for the subjects (Thirion et al., 2014). To overcome these challenges, support vector machines (SVM) have been extensively utilized to manipulate individual brain functional connectivity variation and classify ASD (Yao et al., 2016; Wang et al., 2019). Recently, DL (Deep Learning) approaches have been successfully deployed in neuroimaging research to identify ASD disorder (Li et al., 2018b). Although most of the DL methods used functional connectivity, time-series data analysis, ROI analysis, and spatial or temporal information of fMRI data (Iidaka, 2015; Zhao et al., 2018a), some have issues such as clinical application, lack of model comprehensibility.

As we observed from the recent findings, there are still some drawbacks to overcome in ASD classification using deep learning knowledge, such as lack of data mining techniques from the heterogeneous, complex fMRI data and model interpretation to classify ASD. Besides, a large group of scientists adopted ROIs, or functional connectivity (FC) features to classify ASD. As ASD is heterogeneous, a more pertinent approach is required to classify ASD patients from a typical control. In this paper, we consider a novel DL algorithm for ASD classification to overcome these challenges. The pivotal contributions in this experiment are as follows:

- We preprocessed 3D fMRI data according to the model input requirement through slice-time correction and min-max normalization. We preferred min-max scaling so that data variables can contribute equally and overcome the model biases during training of the classification model.
- We improved the original DarkNet and proposed a novel framework named DarkASDNet for ASD classification. Our proposed framework's main advantage is that it has a fast operating speed and is easily interpretable to weigh against other state-of-the-art methods.
- Finally, to evaluate DarkASDNet performances using the preprocessed fMRI data, we contemplated metrics functioning such as recall, precision, F1-score, and accuracy with ROC curve and AUC score and legitimized our outcomes by distinguishing with recent literature descriptions.

The designed DarkASDNet framework with fMRI processing steps provides a novel benchmark approach for ASD classification on the Autism Brain Imaging Data Exchange (ABIDE) dataset.

## 2. Related Work

The coalescence of brain imaging and machine learning approach concession of ASD classification can alleviate the critical affliction and give precautions to the patient's day-by-day prosperity. Research for brain networks using functional connectivity is a robust method for understanding the neurological bases of various brain disorders, for example, autism (Pascual-Belda et al., 2018). Abraham et al. used a support vector classifier (SVC) in 871 resting-state fMRI data to classify ASD vs. TC and got an accuracy of about 67% (Abraham et al., 2017). According to work in Jin et al. (2015), they proposed the SVM to classify ASD and got the highest accuracy of 76% during testing results with the original multi-kernel.

In Parikh et al. (2019), k-fold cross-validation was promulgated to measure classification performance using specificity, sensitivity, accuracy, and area under the curve (AUC). In Yu et al. (2020), a reverse mapping system was anticipated to further learn reverse mapping to assist mining and representation of task dependencies. Then, an adversarial assumption training approach combined a multi-tasking learning network with a reverse mapping network. Finally, an MRI of the two network parameters learned from the source was shared with target imaging CT (computed tomography). In Zhang et al. (2018), their method treated data at various points in time as different perspectives and built an overarching representation to collect complementary data from the entire time period. The potential representation investigates the complementarity between various time points in order to increase prediction accuracy. The problem is solved using the Alternate Direction Method of Multiplier (ADMM).

For the classification and identification of the regions of interest (ROIs) of functional connectivity magnetic resonance imaging (FC-MRI), Yang et al. (2019) deal with different ML algorithms, including SVM, ridge, and logistic regression where the highest accuracy of 71.98% obtained by ridge classifier. Multiple stacked auto-encoder (SAE) was considered by Guo et al. (2017) as a feature selection technique by ROIs from whole-brain FC. They obtained a classification accuracy of 86.36% utilizing only one data site named UM (University of Michigan) from ABIDE. However, ROIs for the time series data can illustrate and classify ASD from the whole brain. Usually, ROI figures out the functional connectivity pattern and activation of the brain (Eickhoff et al., 2015; Cociu et al., 2018). Dvornek et al. integrated rs-fMRI phenotypic data and obtained an accuracy of 70.1% by deploying LSTM (Long short-term memory) (Dvornek et al., 2018). Without the cross-validation and global signal regression system, they used CCS pipeline data.

In particular, maintaining the 3D and 2D data with the Convolutional Neural Network (CNN) from the DL methods, opens a new era for the classification and segmentation tasks (Parisot et al., 2017; Li et al., 2018a). In order to classify and distinguish ASD from healthy controls, Zhao et al. (2018b) assessed a satisfactory 3D CNN to unite the distinctiveness of functional and spatial brain networks. They integrated only two hundred rs-fMRI (ASD-100, HC-100) data. For the neuropathological biomarker, another way to recognize the brain's patterns is graph convolutional neural networks (G-CNN). Ktena et al. (2017) introduced the connectome-based classification model by applying CNN. Anirudh and Thiagarajan (2019) investigated ensemble learning and G-CNN to classify the problems and achieved 70.86% testing accuracy. Khosla et al. (2018) employed the connectivity fingerprint as a voxel input for the 3D convolutional neural network (CNN) with an accuracy of 73.3% and with ensemble CNN of 75.8%. Wutao et al. learned the features from the raw features by using an autoencoder (AE; Yin et al., 2020). Finally, they amalgamated the pretrained AE and DNN, which leads to an AUC of 82.4% and an accuracy of 79.2%. According to Ahmed et al. (2020), this was performed per site classification to see the data variability. The ABIDE-NYU dataset achieved the highest accuracy of 86 and 88% for stat map and glass brain images, using improved CNN architecture. On the other hand, in Kong et al. (2019), the authors extracted the ROI connectivity features for ASD classification using deep neural network (DNN). They used ABIDE-I, NYU dataset with 10-fold cross-validation. Using the softmax classifier and the stacked autoencoder (SAE) to get the most promising results for the ABIDE-NYU site of 90.39% accuracy according to our best knowledge.

The contemporary scientific knowledge for ASD classification is summarized in Table 1. According to Table 1, it is evident that most of the researcher goes through for classification purposes with functional connectivity (FC) or ROIs data for their work. We noticed there had been an inclination to use machine or deep learning approaches to solve classification problems. In the meantime, with the ABIDE dataset, a preponderance of works focused on a particular atlas, site, or pipeline image to overcome the classification problems. Medical image data are practically preferable to convalesce concrete contributions in the field of brain disorder research like ASD for treatment and reliability (Ravì et al., 2016; Phinyomark et al., 2017). To overcome these challenges, we preprocess every single slice from the whole brain images for each ABIDE-NYU dataset subject. We build DarkASDNet to extract features for the classification problems and check the stability of our model. We executed confusion metrics for precision, recall, F1-score, ROC curve, and AUC value.

**Table 1:** A concise representation of the erstwhile deep learning algorithms in autism classification.

**References**	**Method**	**Pattern**	**Purpose**	**Accuracy (%)**
Leming et al. (2020)	Ensemble learning	FC and structural	Classification	67
Lu et al. (2020)	Auto-encoder	Atlases	Classification	61(ABIDE)
Niu et al. (2020)	DANN	FC/ROI	Classification	73.2
Byeon et al. (2020)	RNN	FC	Classification	74.54
Thomas et al. (2020)	3DCNN, SVM	FC	Classification	66
Jiao et al. (2020)	CapsNets	FC	Classification	71
Yin et al. (2020)	DNN, AE	ROI	Classification	79.2
Anirudh and Thiagarajan (2019)	GCNN	ROI	Classification	70.86
Zhao et al. (2018b)	3D CNN	ICN	Differentiation	70.5
Zhao et al. (2018a)	3D CNN	ROI	Classification	70.1
Guo et al. (2017)	DNN	FC	Classification	86.36
Dvornek et al. (2017)	LSTMs	ROI	Identification	68.5
Ktena et al. (2017)	GCNN	ROI	Classification	62.9
Abraham et al. (2017)	SVM	ROI	Prediction	67

## 3. Materials and Methodology

### 3.1. Dataset

In our experiments, we used ABIDE-I data processed through the Connectome Computation System (CCS) (Craddock et al., 2013). The raw 3D NIFTI fMRI data has been downloaded from ABIDE-I through the CCS pipeline, a publicly available dataset for ASD and TC. Among the 17 sites, we endeavor with the CCS-NYU (New York University Langone Medical Center) site. The publicly available CCS was preprocessed, including a register of the anatomical brain mask to functional image: FLIRT, slice time correction: 3dTshift, Skull-strip: AFNI's 3dAutomask, motion correction: 3dvolreg, voxel intensity normalization, nuisance signal removal, band-pass filtering (0.01–0.1 Hz) [http://preprocessed-connectomes-project.org/abide/ccs.html]. The phenotypic information of the CCS-NYU dataset is shown in Table 2.

**Table 2:** NYU phenotypic data information for ABIDE-I database.

**Total subjects**	**ASD**	**TC**	**Female**	**Male**	**Age range (Years)**		**Average age (SD)**	**ADOS score (SD)**
					**ASD**	**TC**		
184	79	105	35	149	7.1–39.1	6.5–31.8	15.25 (6.58)	11.30 (4.08)

### 3.2. Data Preprocessing

**Figure 2:**
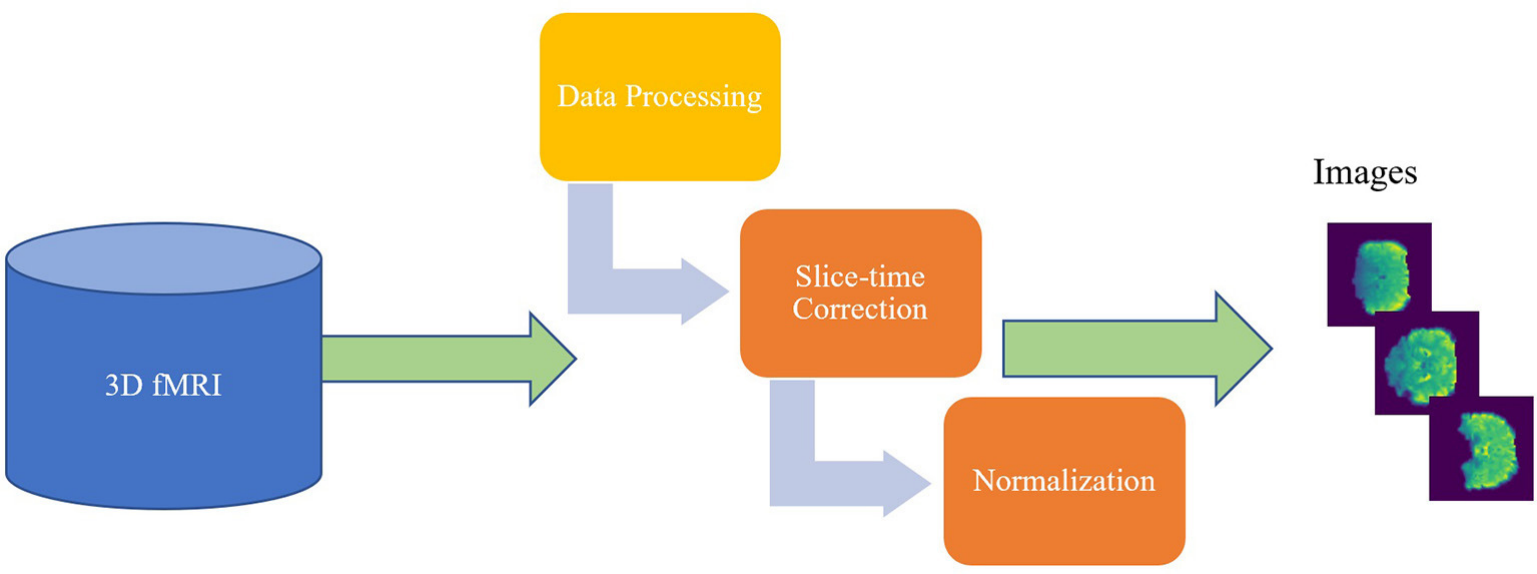
The overview of the 3D fMRI data processing for both ASD and TC.

The CCS ABIDE data preprocessing pipelines are analogous due to the parameters and software used for each of the steps. The CCS parameters and steps are presented in Table 3. In this work, data are selected from the filt_global preprocessing stratagem, which is band-pass filtered (0.01–0.1 Hz) and spatially registered using a nonlinear method to MNI152 template space for each of four pipelines. The overall 3D fMRI data processing procedure is shown in Figure 2. For data processing, firstly, we loaded the 3D fMRI data and saved it as 2D images. To pursue this process, we proceeded with the slice time corrections and normalizations. The whole steps are explained briefly in the following section.

**Table 3:** Overview of the basic parameters and steps of used by CCS.

	**Basic processing**	**Nuisance Signal**	**Regressor Removal**
Steps	Slice timing correction (Yes)	Motion (24 param)	Tissue signals (mean WM and CSF)
	Motion realignment (Yes)		Motion realignment (Yes)
	Intensity normalization (Yes)		Low frequency drifts

#### 3.2.1. Slice-Time Correction

The original 3D fMRI data has 73 slices per volume according to the data description of ABIDE-I. In our experiments, from the 73 slices, we contemplated the last 50 slices because of the precise sketches of the brain images.

#### 3.2.2. Normalization

Normalization is a process wherein the database is reoriented in such a way that users can suitably handle that for further interrogation and analysis. We used the Min-Max normalization technique to overcome the image inappropriateness, which transformed the images into numerical values from 0 to 1.

(1) $$\begin{array}{l} Y_{i}=\left[X_{i}-min\left(X\right)\right]/\left[max\left(X\right)-min\left(X\right)\right] \end{array}$$

Where *X*_*i*_ is the ith data point, min and max stands for minimum and maximum, and *Y*_*i*_ is the converted output.

### 3.3. Proposed DarkASDNet Model

**Figure 3:**
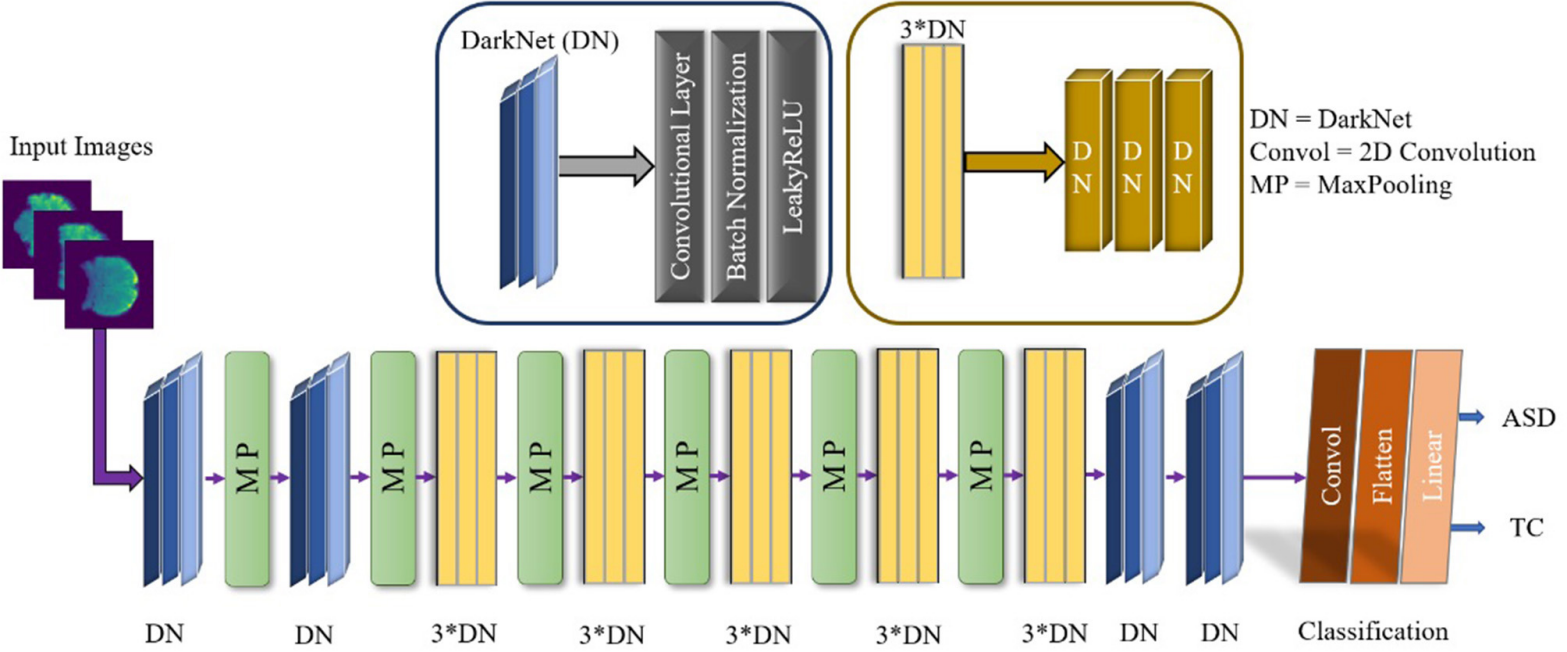
The proposed DarkASDNet framework for ASD classification.

The appositeness of the deep learning approach has to be remolded in artificial intelligence, helping to find neuropsychiatric brain disorders such as ASD. Deep learning is designated with the increasing number of layers as well as the network. An exemplary CNN performed for the feature extraction by the convolution layer, and reduced the size of the computational operation and a fully connected layer before the classification. The overall demonstration of our conceptual DarkASDNet architecture is presented in Figure 3. Here, DN represents the set-up for the convolutional layer, batch normalization layer, and max-pooling layer in sequential order. For the time being, 3^*^DN is betoken for the three times of DN ensuing one after another DN block. There are a considerable number of deep learning algorithms. In this work, we have followed the Darknet-19 model (Redmon and Farhadi, 2017) for our experiment and updated this model to get the utmost accuracy. The Darknet-19 preeminently builds for classifier object detection where they used 19 convolutional layers, 5 Maxpooling layers, and disparate stride values, sizes, and filter numbers. In this work, we proposed DarkASDNet for classifying the autism brain images between ASD vs. TC. For this reason, we have originated 20 Convolutional layers and six Max pooling layers.

Where *Convol* represents the 2D Convolution, and *MP* stand for Max pooling layers. Each Convolutional layer come out with Batch Normalization (BN) and LeakyReLU operations. There has been the same set-up when we are using three Convolutional layers in successive order. For the two-dimensional convolution operation, kernel is epitomized as K and input images as X, while * is symbolized as discrete convolution operation, as given in the following Equation (2).

(2) $$\begin{array}{l} {\left(X*K\right)}_{\left(i,j\right)}=\underset{m}{\sum }\underset{n}{\sum }K_{\left(m,n\right)}X_{\left(i-m,j-n\right)} \end{array}$$

The superiority of adopting batch normalization is increased learning rate, improved gradient flow, reduced dependency on initialization, standardized inputs, and reduced training time to overcome the overfitting problem. Although ReLu (Rectified Linear Unit) or Sigmoid activation functions are prominent in deep learning, we used LeakyReLU as our activation function. Unlike ReLu, LeakyReLU has the biggest advantages in calculating the negative part which forestalls dying neurons. The mathematical formula for LeakyRelu is shown in Equation (3).

(3) $$\begin{array}{l} f\left(x\right)=\{\begin{array}{ll} 0.01x, & \text{for }x<0\\ x, & \text{for }x>0 \end{array} \end{array}$$

Resembling the DarkNet-19, Maxpool has the same operation in our model. It has several advantages, such as reducing the number of parameters to get prime information, diminishing the computational cost, and preventing over-fitting by fixing up with an abstracted form of the depiction. To classify the binary classification problem, we inked the loss function called Cross-Entropy Loss, and for the optimization, we set the Adam optimizer. The main ascendancy of using the Cross-Entropy loss function in binary classification problems is that it can reduce the distance between predicted and actual. The equation for the binary classification of the Cross-Entropy Loss function as follows.

(4) $$\begin{array}{l} CE=-\overset{C^{\prime }=2}{\underset{i=1}{\sum }}t_{i}log\left(s_{i}\right)=-t_{1}log\left(s_{1}\right)-\left(1-t_{1}\right)log\left(1-s_{1}\right) \end{array}$$

where *C*′ = 2 (for two classes *C*_1_ and *C*_2_), *t*_1_[0, 1] and *s*_1_ are the ground truth and score for *C*_1_, *s*_2_ = 1 − *s*_1_ and *t*_2_ = 1 − *t*_1_ for *C*_2_. Finally, the layers and layers parameter are described in Table 4.

**Table 4:** The number of layers and layer parameters of the proposed DarkASDNet model.

**Layer**		
**(type)**	**Output shape**	**Parameters**
Convol2d	[8, 256, 256]	216
Convol2d	[16, 128, 128]	1,152
Convol2d	[32, 64, 64]	4,608
Convol2d	[16, 66, 66]	512
Convol2d	[32, 66, 66]	4,608
Convol2d	[64, 33, 33]	18,432
Convol2d	[32, 35, 35]	2,048
Convol2d	[64, 35, 35]	18,432
Convol2d	[128, 17, 17]	73,728
Convol2d	[64, 19, 19]	8,192
Convol2d	[128, 19, 19]	73,728
Convol2d	[256, 9, 9]	294,912
Convol2d	[128, 11, 11]	32,768
Convol2d	[256, 11, 11]	294,912
Convol2d	[512, 5, 5]	1,179,648
Convol2d	[256, 7, 7]	131,072
Convol2d	[512, 7, 7]	1,179,648
Convol2d	[256, 9, 9]	131,072
Convol2d	[512, 9, 9]	1,179,648
Convol2d	[2, 9, 9]	9,216
Flatten	[162]	0
Linear	[2]	326

We utilized the Cross-Entropy loss function with the Linear classifier because they are best fitted to our proposed binary ASD classification instead of preserving the original DarkNet's loss calculation strategy. In the meantime, we change the average pooling layer to maxpooling layer and add one more convolutional layer than DarkNet. Moreover, the trainable parameters of the proposed DarkASDNet model are about 4.5 million compared to the underlying DarkNet model, which contains around 25 million. Therefore, our model is six times lighter than the original DarkNet, ensuring our model's computational efficiency.

## 4. Data Visualization and Performance Metrics

### 4.1. Visualization of the Sliced fMRI Data

**Figure 4:**
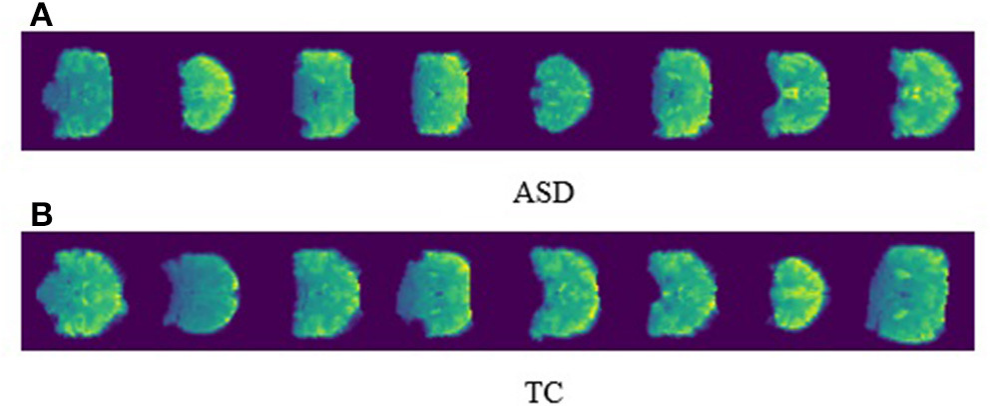
The visualization of single sliced ASD and TC images.

For the outrun treatment, reinforcement with prior diagnosis is important for ASD patients in order to delay deterioration and retain quality of life The visualization of the neuroimaging data can outrun perceptible biomarkers to illustrate prognosis and particular pathology for ASD patients. In our proposed work, we preprocess the 3D fMRI data into 2D images with slice time correction and normalization. Figure 4 represents the perfect visualization of our preprocessed images, and the manifestation of our per slice images are easily depicted for both ASD and TC. In the meantime, we disclosed only the first eight images for ASD and TC.

### 4.2. Evaluation Metrics

To ensure the performance of our proposed model, we consummate an in-depth search to learn hyperparameters and investigate the average accuracy, f1-score, precision, and recall. True positive (TP) can correctly predict the ASD class and true negative (TN) for TC. False positive (FP) is the outcome of incorrect prediction of ASD and false negative (FN) for TC. The corresponding formula for the evaluation metrics is given below.

(5) $$\begin{array}{l} Accuracy=\frac{Total\;correct\;prediction}{Total\;number\;of\;labels}\times 100 \end{array}$$

(6) $$\begin{array}{l} Precision=\frac{True\;Positive}{True\;Positive\;+\;False\;Positive} \end{array}$$

(7) $$\begin{array}{l} Recall=\frac{True\;Positive}{True\;Positive+False\;Negative} \end{array}$$

(8) $$\begin{array}{l} F_{1_{-}\text{score}}=2\times \frac{\left(Precision\;*\;Recall\right)}{\left(Precision\;+\;Recall\right)} \end{array}$$

## 5. Experimental Results and Discussion

**Figure 5:**
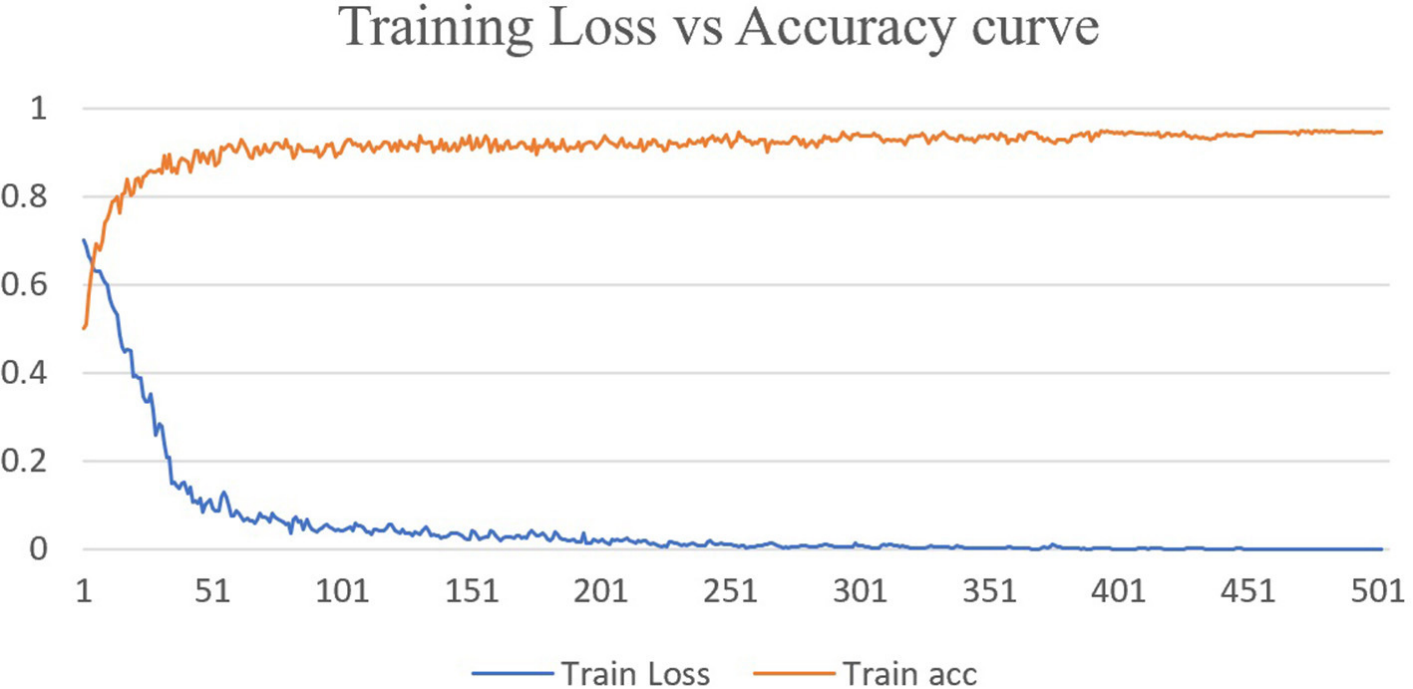
Training loss vs. accuracy curve for DarkASDNet.

**Figure 6:**
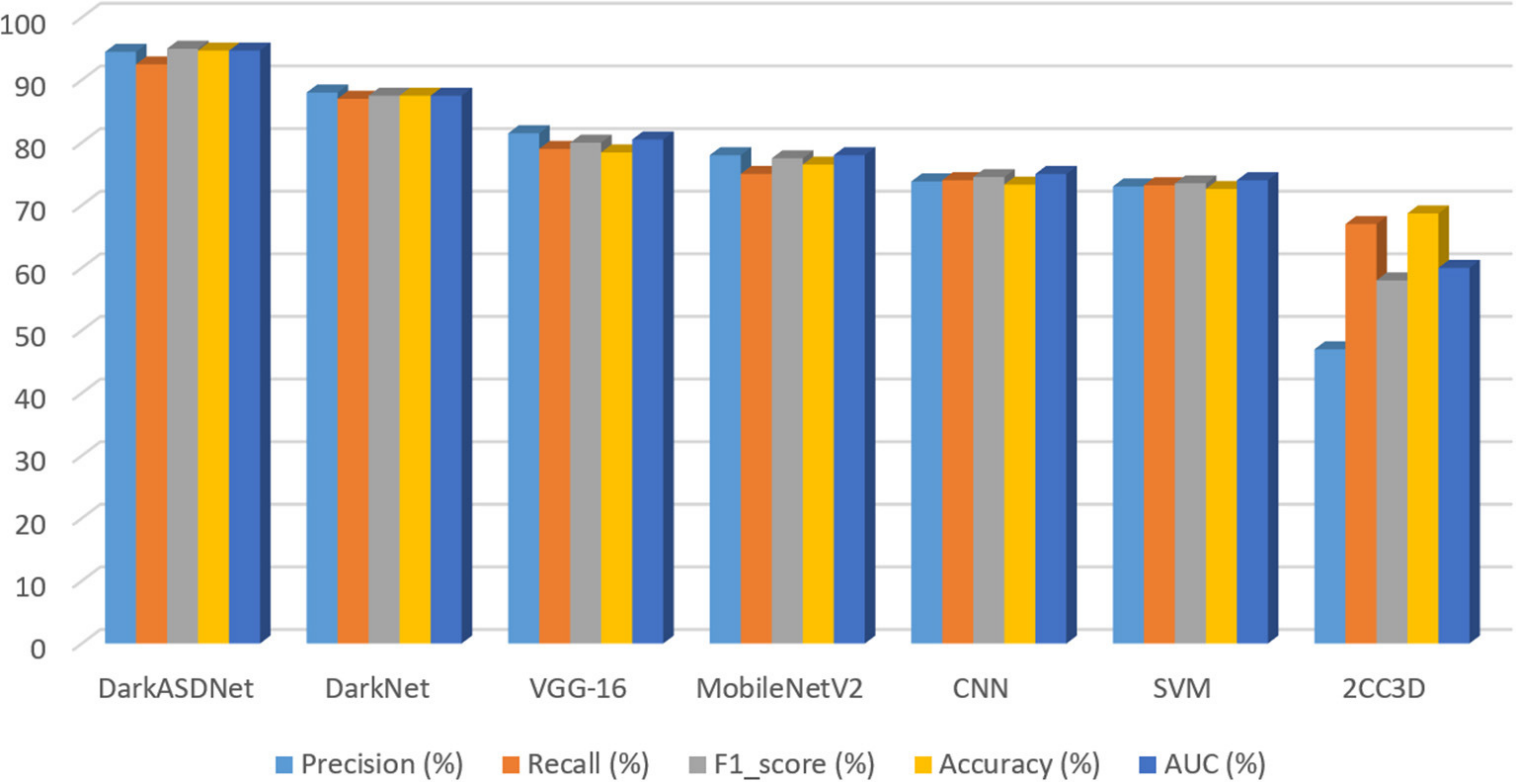
Accuracy comparison of DarkASDNet with state-of-the-art methods used in ASD classification.

Deep learning approaches have been successfully employed in ASD classification using ABIDE data based on fMRI images. In this work, we have proposed DarkASDNet to classify ASD showing different measurement metrics in the same manner with recall, precision, F1-score, and accuracy with ROC curve and AUC score to legitimize the performance of the proposed method. The training loss and accuracy comparison curve of the proposed DarkASDNet is shown in Figure 5. From Figure 5, it is depicting that the training accuracy is increasing with decreasing the training loss. The highest accuracy of 94.7% we have obtained while testing the DarkASDNet model. Figure 6 shows the performance values for different evaluation metrics, including precision, recall, f1-score, and AUC for ASD classification using proposed DarkASDNet. After testing our model for ASD classification, we have achieved the highest accuracy of 94.7%, the precision of 94.5%, recall of 92.5%, f1-score of 95%, and the AUC score 94.703%.

Furthermore, to evaluate our proposed DarkASDNet, we have implemented the VGG16 (Simonyan and Zisserman, 2015), MobileNetV2 (Sandler et al., 2018), and SVM (Jebapriya et al., 2019) algorithms as competence methods to classify ASD using the same dataset. We have also implemented two most recent works preferably using CNN in Ahmed et al. (2020), and 2CC3D in Li et al. (2018a). Figure 6 represents the performance comparison for the competence method with our proposed DarkASDNet model. Using SVM, MobileNetV2, and VGG16 models for ASD classification, we get an accuracy of 72.6, 76.5, and 78.43%, respectively. Comparing with the performance of competitive methods, in DarkASDNet, we get state-of-the-art accuracy during classification. As we know, deep learning techniques are nowadays performing very satisfactorily in ASD classification using ABIDE data. For example, in Ahmed et al. (2020), authors adopted the CNN model for single site (NYU) classification, where they got the highest accuracy of 88% for glass brain images. On the other hand, using extracted ROIs connectivity features and NYU dataset with 10-fold cross-validation, Kong et al. (2019) achieved the highest accuracy of 90.39% by considering stacked autoencoder (SAE) and softmax as a classifier. Furthermore, Auto-ASD-Network proposed by Eslami and Saeed (2019) based on the multilayer perceptron (MLP) with two hidden layers, and SVM got the highest accuracy of 80% for ASD classification using the NYU dataset. The accuracy comparison curve of our proposed DarkASDNet and other state-of-the-art methods for ASD classification using the ABIDE-NYU dataset is shown in Figure 6. From the bar diagram in Figure 6, the nearest model has a mean accuracy difference of about 3.4% with our method (Kong et al., 2019). Therefore, based on the results, our proposed DarkASDNet method outperforms other methods on average for classifying ASD.

**Figure 7:**
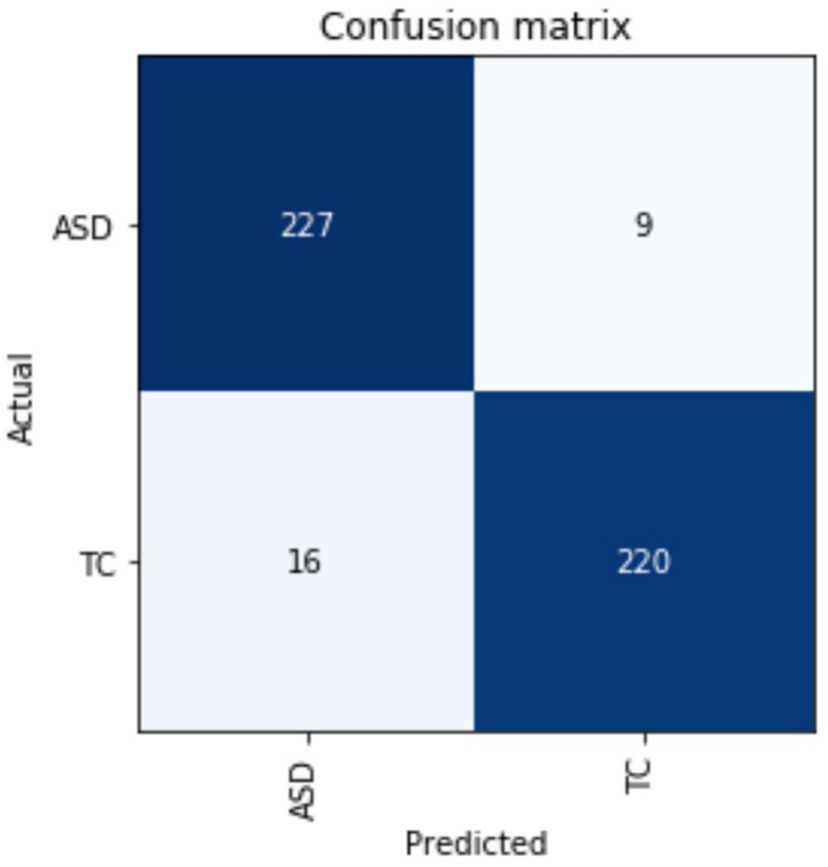
The evaluation performances of confusion metrics.

**Figure 8:**
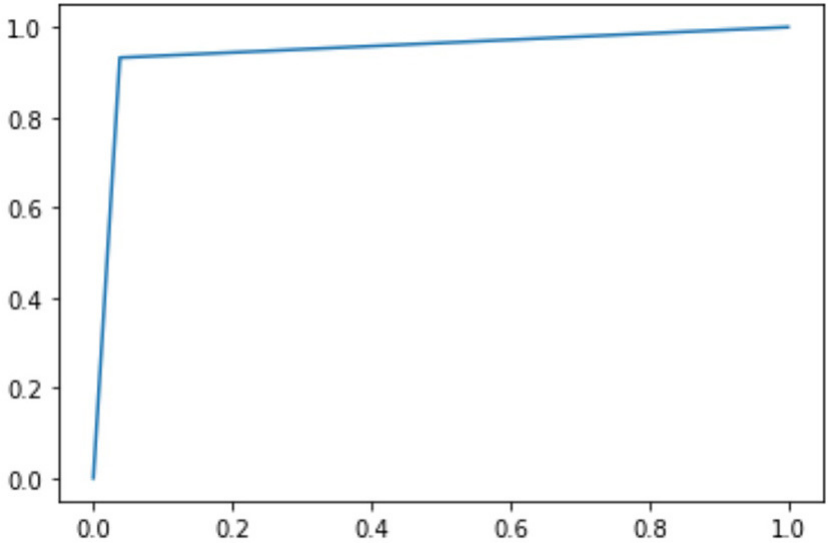
Receiver operating characteristic curve for ASD.

Besides the matrices explained above, we have also described the confusion matrix table for DarkASDNet in Figure 7. It is clear from Figure 7 that there are two predicted classes: ASD and TC, a binary classification problem. The proposed DarkASDNet classifier made 472 subjective predictions, and out of these subjects, the classifier predicts 243 times as ASD patients and 229 times as the TC subjects. However, in the original dataset, there were 236 subjects for ASD patients and 236 subjects as TC. From the confusion matrix, we see that the overall misclassification rate is ~5.3%, with a true positive and false positive rate of 0.96 and 0.93, respectively, which is comparatively highly acceptable. The corresponding ROC curve is shown in Figure 8.

## 6. Conclusion and Future Work

It is challenging to find the proficient classifier for ASD, while most of the classifier depends on functional connectivity and brain ROIs analysis. In this work, we proposed a novel DarkASDNet model for ASD classification using 3D fMRI data. Different from the conventional machine learning method in which the extraction of the image features for the training set is done manually, our method handles the extraction of the image features automatically during the computation. We processed the fMRI data according to the DarkASDNet requirement through proper slice-time correction and normalization. We assessed the DarkASDNet performances using the generated fMRI data and utilized metrics functioning as recall, precision, F1-score, and accuracy with ROC curve and AUC value. Finally, we validated our outcomes by comparing with five other recent competency methods, including three leading benchmark approaches showing state-of-the-art results. To the end, the proposed framework provides a new benchmark method for ASD classification.

Future work will increase the number of subjects, such as the whole ABIDE database, considering each subject's phenotypic information. Although our model has presented outstanding results for ASD classification, improvements still need to be made to the model to handle the 3D fMRI data directly. We will solve these issues in our future work by employing the sample demographic information.

## Data Availability Statement

The raw data supporting the conclusions of this article will be made available by the authors, without undue reservation.

## Author Contributions

MSA and SN conceived the study. MSA pursued the implementation of the methodology, conducted the data processing and experiments, and generated and validated the results. MSA, SN, and MRA performed the formal analysis and wrote the original draft. SN provided critical feedback and suggestions for performing the experiments. SN, MRA, JD, XG, and YC provided valuable suggestions in writing the manuscript. All authors have read and agreed to the published version of the manuscript.

## Conflict of Interest

The authors declare that the research was conducted in the absence of any commercial or financial relationships that could be construed as a potential conflict of interest.
